# Socio-Demographic, Professional and Institutional Characteristics That Make Romanian Doctors More Prone to Malpractice Complaints

**DOI:** 10.3390/medicina58020287

**Published:** 2022-02-14

**Authors:** Bianca Hanganu, Magdalena Iorga, Lavinia Maria Pop, Beatrice Gabriela Ioan

**Affiliations:** 1 Legal-Medicine Department, Faculty of Medicine, “Grigore T. Popa” University of Medicine and Pharmacy of Iasi, 700115 Iasi, Romania; bianca-hanganu@umfiasi.ro (B.H.); beatrice.ioan@umfiasi.ro (B.G.I.); 2 Behavioral Sciences Department, Faculty of Medicine, “Grigore T. Popa” University of Medicine and Pharmacy of Iasi, 700115 Iasi, Romania; 3 Faculty of Psychology and Education Sciences, “Alexandru Ioan Cuza” University of Iasi, 700554 Iasi, Romania; lavinia-maria.pop@umfiasi.ro

**Keywords:** medical malpractice, complaints, doctor, Romania, socio-demographic characteristics, professional characteristics, institutional characteristics

## Abstract

*Background and objectives*: Medical malpractice is a phenomenon that shadows current medical practice, the number of complaints following an upward trend worldwide. The background for complaints is related both to the doctor and medical practice in general, as well as to the patient. The aim of this study was to identify a profile of the Romanian doctors who are more prone to receiving complaints, by analyzing the socio-demographic, professional and institutional characteristics. *Materials and Methods*: We conducted a quantitative, prospective research, the data being collected using a newly developed questionnaire. Data analysis was performed with the IBM Statistical Package for Social Sciences (SPSS, version 24). We used counts, percentages, means and standard deviation, and comparative and correlational analyses. A logistic regression model was applied to select a statistically best-fit model to identify independent predictors for receiving complaints; a Hosmer–Lemeshow test was used to check the performance of the prediction model. *Results*: The study group consisted of 1684 doctors, of which 16.1% had been involved in a malpractice complaint. Results showed that men, senior doctors from surgical specialties who perform a greater number of on-call shifts, those who work in regional or county hospitals, those who have greater fear of receiving complaints and those whose life partner is a doctor with the same specialty are more prone to receiving complaints. *Conclusions*: The profile identified by the present research underlines the main characteristics that could be targeted with specific measures in order to prevent the ongoing increase of malpractice complaints in Romania.

## 1. Introduction

The need to respect the norms of good practice in medicine has accompanied the medical profession throughout its evolution, but the perspective on failure in the medical act has undergone important changes over time. In general, complaints regarding medical professional liability are based on medical error: real, or perceived as such by the patient or his/her family.

The large number of complaints about medical professional liability all over the world is a reality that cannot be disputed, and the numerous consequences they generate sound the alarm about the need to identify prevention and reduction strategies. The data published in the literature provide an overview of the number of complaints in different countries of the world. For example, the analysis conducted in 2016 by Guardado [1] shows that 34% of members of the American Society of Medicine received at least one complaint during their career, and almost half of them (16.8%) faced at least two complaints. In Europe, Italy was the country with the highest number of complaints in 2009 [2], with an increase of 255% from 1994 to 2011 [3] and a total of about 16,000 complaints made annually by patients [4]. A study conducted in Saudi Arabia shows a tripling of the number of complaints between 1999 and 2008, with an increase from 440 to 1356 over the ten years of the study [5]. An analysis of the annual distribution of malpractice complaints filed in Romanian courts between November 2007 and April 2018 shows an increase in the number of complaints from 8 in 2008 to 65 in 2017, with 331 complaints filed during the entire study period [6]. The analysis of the complaints submitted for extra-judicial analysis in the eight counties of the Moldova region of Romania shows a total of 153 complaints in the period 2006–2019 [7].

The natural reaction of patients to the real or perceived failure of a medical procedure is the search for the culprit, and their tendency—sometimes wrong—is to put full responsibility on the shoulders of the attending physician [8]. Analyzing the human factor in medical errors, the scientific community agrees that the risk of error exists and hovers above even the most experienced physicians [9], as failure may occur despite compliance with all rules of good professional practice [10]. Failure in medical practice is often the result of complex interactions that go beyond the individual limit of the doctor involved. Thus, it is about a chain of elements that include the doctor, the institution in which he/she works, the medical system [11], the patient and the medical science itself, with its inherent limits.

Any medical act has an intrinsic risk, accepted by the medical community, about which the patient must be informed and which he/she in turn accepts once the informed consent form is signed. However, patients cannot always distinguish between the situations in which the doctor was wrong and the situations in which the failure occurred despite the fact that the doctor followed exactly the specific conduct of that medical procedure [10].

The decision of the patient or his/her family to file a complaint against the doctor is based on a combination of factors, among which the characteristics of the doctor play an important role. Thus, the data reported in the literature show that the risk of a doctor for being reported depends, on the one hand, on socio-demographic factors, such as age [1,12,13], gender [1,13] or place of birth [14], and on the other hand on educational and professional factors, such as: specialty [1,12,13], seniority in work [5,13], number of patients [15] and their level of satisfaction [15], type of medical educational institution from which they graduated (public or private), whether or not they took postgraduate training courses [5,13], the number of days off [12], the volume and location of the medical office [5], and academic activity [16]. In addition, the existence of one or more previous claims contributes to the doctor’s increased risk of receiving a complaint [17].

Knowledge of the characteristics of doctors complained about by patients and the factors that influence these characteristics can be the foundation for complaints prevention strategies focused on the changeable factors, regarding both the doctor–patient relationship and the characteristics of the health care system, in terms of better cooperation between different levels of the health care system and in-depth analysis of the reasons that lead to complaints in specialties with a higher risk of complaints.

The aim of this study was to identify the socio-demographic, professional and institutional characteristics profiling the Romanian doctor prone to receiving complaints, by analyzing the characteristics of doctors involved in complaints made by patients in Romania.

## 2. Materials and Methods

### 2.1. Instruments and Data Collection

For the construction of the questionnaire, a literature data search was undertaken and supplementary information about items was suggested by the results of previous studies by the present research team: a retrospective study regarding patient complaints submitted to the Commission for monitoring and professional competence for malpractice cases within the Public Health Directorates in the Moldova region of Romania [7] and a qualitative study based on semi-structured interviews with doctors who were complained about by patients.

The questionnaire was evaluated by five experts from different specialties and was pre-tested before being distributed, with the corresponding improvements implemented based on the feedback received. The final form was approved by the research team.

A total of 88 questions were structured within three main sections: the first part included 44 questions that were addressed to all doctors, the second part included questions intended for doctors who know a colleague who was complained about, and the third part included questions for doctors who themselves were involved in a complaint from patients. The separation of the sections and the categorization of the participants regarding whether they were the subject of a complaint from a patient were made according to the answers to the question as to whether they know anyone who had received a complaint from a patient, with the following possible answers: a colleague, me, no one. In this paper we present the results of the analysis of the first part. The majority of answers were collected using a Likert-like scale and some of the items were open.

Accordingly, the data collected for this study were obtained from the questions concerning the following issues: the characteristic of being the subject of a complaint or not, socio-demographic data (such as age, gender, marital status, profession of the life partner, parenthood, working area), professional and institutional information (specialty, professional degree, seniority in the medical profession and at the current workplace, the type of medical institution in which they work, the number of work places, the number of on-call shifts, the academic degree, the type of employment contract and the performance of the full time job in a medical education institution, the type of medical education institution they graduated, holding a management position, number and type of patients they examine), characteristics related to the process of continuing medical education (participation in national/international congresses/conferences, certifications/competencies, postgraduate courses), level of personal and professional satisfaction (relationship with family, colleagues, superiors, social life, working conditions, facilities of the institution where they work, workload, financial situation, rest time), level of concern about the occurrence of adverse events (death of the patient, complications of the investigations, intraoperative and postoperative complications, adverse drug effects), characteristics connected to the relationship with patients (explaining the information from the informed consent form, explaining the results of tests, patience with difficult patients, limiting information to elderly patients, providing educational materials, offering the possibility for out-of-hours contact, consultation with colleagues in difficult cases), circumstances for disclosing the occurrence of an adverse event (never, always or depending on the severity: minor/serious) and the fear of being complained about.

### 2.2. Participants

The questionnaire was distributed online, using Google Docs, by the College of Physicians, to all the doctors in Romania, members of the College of Physicians, and was opened for three weeks, between 4 May and 24 May 2020. On the first page of the questionnaire the participants found a detailed presentation of the aim and objectives of the study, and a notice regarding the scientific use of the information provided and the confidentiality of data, also stating that participation was anonymous and voluntary.

### 2.3. Statistical Analysis

Data analysis was performed with the Statistical Package for Social Sciences (SPSS, version 24, Armonk, NY, USA). Descriptive analysis presented percentages, means and standard deviation to describe the variables. For comparative analysis a Mann–Whitney rank statistic test was performed in order to detect and describe significant differences between variables. The correlation analysis of data was done using Spearman correlation. A binomial logistic regression model was used in order to select a statistically best-fit model to identify independent predictors for being complained about. We used the Hosmer–Lemeshow test to check the performance of the prediction model. The level of statistical significance was set at *p* < 0.05.

### 2.4. Ethical Approval

The study was approved by the Research Ethics Commission of the Grigore T. Popa University of Medicine and Pharmacy of Iasi, Romania, registered as No. 16434/30 July 2019.

## 3. Results

### 3.1. Socio-Demographic and Professional Data

**Figure 1:**
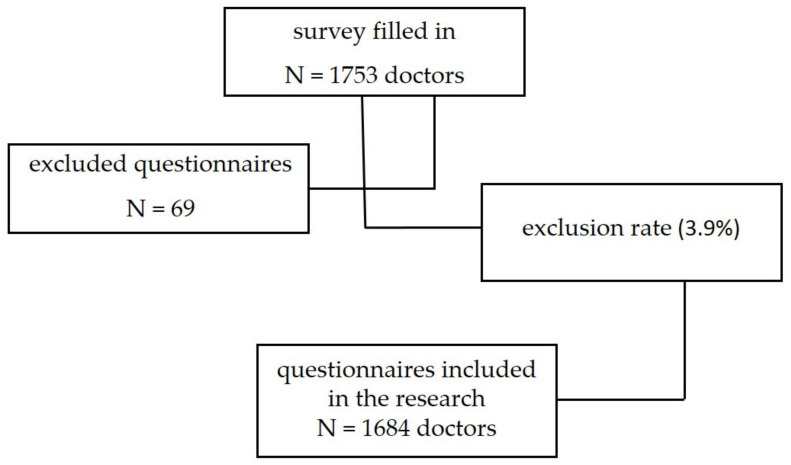
Study profile.

The questionnaire was filled in by 1753 doctors. Of the total number of questionnaires received, 3.9% (N = 69) were excluded from the study because they were not fully filled in. Figure 1 provides details on the response rate.

The analysis of the socio-demographic data indicated that 71.0% (N = 1196) of the participants were women; the mean age of the participants was M = 44.77 ± 10.98 years.

The research was attended by senior doctors (52.1%, N = 878), specialists (38.3%, N = 645) and residents (9.6%, N = 161). A majority of participants, 90.4% (N = 1522), practiced only in urban areas, with a length of employment in the medical system of M = 18.09 ± 11.53 years, and 16.1% of doctors (N = 271) declared that they had experienced complaints from their patients during their career.

Of the total number of doctors included in the research, just over half of them said they had more than one job (50.3%, N = 847). On average, a doctor provided medical services for a number of M = 69.20 ± 57.22 patients per week. The analysis of data showed that the number of on-call shifts/month was M = 2.26 ± 2.78.

A total of 243 doctors (14.4%) were also teaching in medical universities and 27.7% (N = 466) of the participants declared that they held a management position.

According to the nomenclature of medical specialties in the Romanian healthcare system, five categories of specialties were considered: surgical specialties, medical specialties, paraclinical-laboratory specialties, pediatric specialties (pediatrics, neonatology, pediatric surgery and orthopedics) and family medicine, and the presentation of data took these criteria into consideration. Detailed socio-demographic and professional characteristics of the participants are presented in Table 1.

### 3.2. Comparative Analysis

Results of the Mann–Whitney test showed that there were significant differences depending on gender (z = −7.960, *p* < 0.001) and parental status (z = −3.317, *p* = 0.001) when we compared doctors who were/were not involved in malpractice complaints. This means that male doctors (Mean rank = 936.48) face complaints of malpractice more often than female doctors (Mean rank = 804.15). In addition, subjects who were parents declared to a higher extent (Mean rank = 859.76) that they experienced malpractice complaints than doctors without children (Mean rank = 806.24). Significant differences were also recorded in terms of partner profession (z = −3.615, *p* < 0.001) meaning that subjects whose partner was a doctor of the same specialty had received complaints of malpractice more often (Mean rank = 532.10) than subjects whose partner had another profession that was not related to the medical system (Mean rank = 466.88).

When analyzing the categories of medical specialties that had the highest risk of receiving complaints related to malpractice, the results showed that surgical specialties have the highest risk. Thus, the results of the Mann–Whitney test (z = −6.369, *p* < 0.001) showed that doctors specializing in family medicine (Mean rank = 311.06) have a lower risk of having complaints related to malpractice compared with doctors working in the field of surgery (Mean rank = 376.31). Also, there are significant differences between the specializations of surgery and pediatrics (z = −3.031, *p* = 0.002) in the sense that doctors specializing in pediatrics (Mean rank = 222.07) are less likely to be reported compared with doctors in the field of surgery (Mean rank = 255.29). Moreover, the results showed that (z = −3.763, *p* < 0.001) doctors working in the field of surgery (Mean rank = 287.29) are more likely to receive complaints related to malpractice even than doctors working in the laboratory (Mean rank = 247.90).

There are significant differences between the professional degree of doctors in terms of receiving malpractice complaints (z = −6.560, *p* < 0.001) in the sense that senior doctors are more likely to receive such complaints (Mean rank = 538.25) as opposed to resident doctors (Mean rank = 420.45). There are also significant differences between specialist and resident doctors (z = −3.390, *p* = 0.001) in the sense that specialist doctors (Mean rank = 409.86) are more likely to receive malpractice complaints as opposed to resident doctors (Mean rank = 378.01). Moreover, the results (z = −7.466, *p* < 0.001) showed that senior doctors (Mean rank = 809.64) are more likely to be sued for malpractice as opposed to specialist doctors (Mean rank = 697.16).

The Mann–Whitney test results showed that there are significant differences (z = −4.150, *p* < 0.001) in receiving malpractice complaints among doctors, depending on the type of institution in which they work, meaning that doctors working in regional or county hospitals receive more malpractice complaints (Mean rank = 879.20) than those working in smaller hospitals (Mean rank = 815.81). Data also showed that (z = −2.325, *p* = 0.020) doctors working in a hospital or private clinic (Mean rank = 864.40) are more likely to receive malpractice complaints than doctors who do not work in the private hospital environment (Mean rank = 828.46).

Significant differences (z = −2.810, *p* = 0.005) exist between the status of university teachers regarding the existence of malpractice complaints, in the sense that doctors who are at the same time teachers are more likely to receive malpractice complaints (Mean rank = 894.11) unlike doctors who do not teach at university (Mean rank = 833.80). Moreover, the results of the Mann–Whitney test (z = −2.347, *p* = 0.019) showed that doctors who are employed full time in a higher medical education unit (Mean rank = 884.79) are more likely to receive complaints related to malpractice than doctors who do not work full time in such an institution (Mean rank = 835.09).

Doctors were asked to rate their fear of being complained about from 1 (very much) to 5 (not at all). The comparative analysis (z = −6.127, *p* < 0.001) showed that the fear of complaints score is significantly higher for those who have not received any malpractice complaints (Mean rank = 872.88) compared with those who have received malpractice complaints (Mean rank = 684.10).

### 3.3. Correlational Results

The correlation analysis showed that there are positive correlations between the existence of a complaint among the doctors included in the research and various socio-demographic variables. It has been identified that age is positively correlated with the existence of a complaint (*p* < 0.001, r = 0.194), in the sense that as doctors age, the likelihood of dealing with malpractice complaints increases. Seniority in the medical system is positively correlated with the existence of a malpractice complaint (*p* < 0.000, r = 0.202), identifying the fact that a large number of years spent in the medical system increases the probability of r malpractice complaints among doctors. Also, the professional degree held by doctors is positively correlated with the existence of complaints (*p* < 0.001, r = 0.230), in the sense that the higher the professional degree, the higher the probability of malpractice complaints.

Positive correlations were identified between the existence of malpractice complaints and the number of on-call shifts performed monthly (*p* = 0.001, r = 0.079), in the sense that the higher the number of on-call shifts per month, the higher the likelihood of dealing with malpractice complaints. The explanation by the doctors of the results of the interventions and of the performed procedures is positively correlated with the existence of malpractice complaints (*p* < 0.001, r = 0.101), which means that the incomplete explanation or lack of explanation of these results can increase the probability of a malpractice complaint being filed. In addition, patience with difficult patients (*p* = 0.017, r = 0.058) and the distribution of educational materials among them (*p* = 0.024, r = 0.054) are positively correlated with the risk of being sued for malpractice, in the sense that as the patience of doctors decreases and the number of educational materials distributed is lower or even absent, the risk of doctors being sued for malpractice increases.

Spearman correlation analysis indicates that the fear of complaints is negatively correlated with the existence of a complaint related to malpractice (*p* < 0.001, r = −0.049) in the sense that the less worried doctors are that they might be sued, the lower the likelihood of malpractice complaints.

### 3.4. Assessing the Combined Effects of the Factors That Predispose to the Complaint

In order to highlight the strongest probability factors for receiving a complaint, we performed a logistic regression analysis on the characteristics of being complained about, including as predictors age, gender, parental status, professional degree, partners’ profession, specialty of doctors, seniority in the medical system, the number of on-call shifts performed per month, the fear of being complained about, working in a regional/county hospital and university teacher status. The method selected for the logistic regression was the Enter method, analyzing the factorial variables simultaneously. Linearity of the continuous variables with respect to the logit of the dependent variable was assessed via the Box–Tidwell (1962) procedure. Based on this assessment, all continuous independent variables were found to be linearly related to the logit of the dependent variable. 

Table 2 shows the results of the Omnibus test for the model coefficients. The results of the test χ^2^ and of the likelihood rate—2LL recorded in step 1 compared with the initial step 0 allow us to reject the null hypothesis and to accept the alternative hypothesis.

Table 3 shows the results of Hosmer–Lemeshow test. This divides subjects at decile level, based on the estimated probabilities, applying in the next step the test χ^2^ on the frequencies noticed. The *p* = 0.473 value indicates that the logistic model is valid from a statistical point of view, and therefore the null hypothesis can be rejected.

Table 4 shows the estimated values of regression coefficients of the model of logistic regression. Sig. values *p* < 0.05 showed that some factorial variables of the regression model are significant from a statistical point of view and that they influenced the status of receipt of complaints. Similarly, the Wald test values showed that the regression parameters B are different from zero. Therefore, the null hypothesis is rejected. These estimated values of the regression coefficients showed the relation between factorial variables and the dependent variable “complained”: it increases (or decreases, if the coefficient sign is negative) the determined value log odds of the variable of confirmed = 1 at a change with one unit of one of the factorial variables. The influence of the other factorial is considered to be constant.

The model explained 24.6% (Nagelkerke R2) of the variance in complaints and correctly classified 85.5% of cases. Sensitivity was 20.1% and specificity was 98.1%. Of the eleven predictor variables, seven were statistically significant: gender, specialty category, professional degree, partner’s profession, fear of complaints, number of on-call shifts monthly and working in a regional hospital (as shown in Table 4). Women had 0.39 times lower odds of receiving malpractice complaints than men. An increased number of on-call shifts was associated with an increased likelihood of receiving malpractice complaints and a low level of fear about the risk of being complained about is associated with a lower likelihood of facing malpractice complaints. In addition, doctors working in regional hospitals had 1.40 times higher odds of experiencing malpractice complaints.

Professional degree is represented by two dummy variables. The first dummy variable is a comparison of specialist and resident doctor groups. The positive coefficient suggests that specialist doctors had 6.85 times higher odds of being sued for malpractice than resident doctors. Similarly, the second dummy variable compares senior doctors to residents, with results showing that senior doctors had 16.32 times higher odds of being reported for malpractice than resident doctors.

The specialty category of doctors is represented by four dummy variables. The first dummy variable is a comparison of medical specialties with those of family medicine, with the results suggesting that doctors who practice medical specialties had 2.09 times higher odds of being reported for malpractice than doctors in the family medicine specialty. Similarly, the results show that doctors in surgical specialties had 3.15 times higher odds of being reported for malpractice than doctors in family medicine.

Although the partner’s profession is represented by five dummy variables, only two of them show significant differences. Thus, the negative coefficients show that doctors who have a partner who practices a different specialty of medicine had 0.44 times lower odds of being sued for malpractice than doctors who have a partner who practices the same medical specialty as them. In addition, doctors who have a partner who does not work in the medical system had 0.55 times lower odds of being sued for malpractice than doctors whose partner practices the same medical specialty as them.

## 4. Discussion

The present study involved a total of 1684 doctors, of whom 16.1% were complained about at least once in their careers. The binary logistic regression showed that the doctors prone to being complained about are men, with senior doctor degree, from surgical specialties, who perform a greater number of on-call shifts, who work in regional or county hospitals, who have greater fear of being complained about and whose life partner is a doctor with the same specialty.

Regarding the gender of the doctor, both the present study and other studies identified in the literature—both older [16,18,19] and more recent [1,17,20,21]—place men at higher risk for being the subject of patients’ complaints compared with women. The study conducted by Guardado [1] shows that 40% of male doctors and 22.8% of female doctors who were members of the American Medical Association faced a complaint of malpractice during their career, with 20.4% of male doctors being complained about at least twice, compared with 9.7% of female doctors. In the study performed by Tibble et al. [20], focused on surgery, it was reported that male surgeons were 1.31 times more likely to be exposed to a complaint compared with their female colleagues.

There are multiple explanations for this finding. For example, Guardado [1] analyzed the gender of doctors who received complaints in terms of age and specialty and observed after separate analyses that the women were younger than the men, which means less seniority, less experience and consequently a shorter period of time in which they were exposed to the risk of complaints compared with their male colleagues. Likewise, the same author noted that except for obstetrics & gynecology, one of the specialties recognized as frequently complained about, women had specialties in which the risk of being complained about is generally low (e.g., pediatrics, psychiatry). In the same context, Taragin et al. [16] noted that male doctors, by the nature of their specialties, have a higher risk of being complained about because they deal with more severe cases.

A more in-depth analysis identifies additional explanations for men’s predisposition to be complained about and implicitly for the lower risk of complaints received by women. Thus, Hall et al. [22] and Fountain [23] link the gender of doctors with their skills regarding the doctor–patient relationship and report that women have the advantage of interacting with patients more effectively, in a manner characterized by less hostility, being more meticulous, having more humanistic attributes [22], and having greater emotional involvement and a more positive approach to the patient [22,23]. Added to these are the differences in communication style [23], women more often adopting patient-centered communication, in which the patient is actively involved in the decision-making process [22,23], and often providing psycho-emotional counseling [23] and devoting more time to the discussion with the patient [22,23]. The time allotted for the discussion with the patient can be viewed from two perspectives: on the one hand, when the doctor spends more time with the patient, the latter will have the opportunity to express his/her concerns and will be more satisfied with his/her doctor. In this sense, Taragin et al. [16] argue that when patients are satisfied with the relationship they have with their doctors, they are less tempted to complain when the result of the medical procedure does not coincide with expectations. On the other hand, the time spent in discussion with the patient allows the consultation of a smaller number of patients and thus the exposure to a lower or higher risk of being complained about, respectively [22].

Although in the present study the binary logistic regression excluded older age and higher seniority from the initial profile, these two characteristics can be related to the result that identifies the degree of senior doctors as a predisposition to receiving complaints. The first stage in the doctor’s training is the residency, which in Romania lasts between three and six years, depending on the specialty [24]. Next is the degree of specialist doctor, which is obtained after an examination, at the end of the residency period [25]. The degree of senior doctor is not a mandatory step in the career of doctors, but most doctors choose to sustain the examination for this promotion. In order to sustain the examination to become a senior doctor, the candidate must have sufficient experience as a specialist, practicing for at least five years in the specialty [26]. As other studies published in the literature point out, doctors’ risk of facing a patient complaint increases in direct proportion to age [1,16,18] and implicitly to seniority [13,27]. Guardado [1] found that of the total number of doctors under the age of 40, 8.2% had faced a complaint during their career, whereas among doctors over the age of 54, the percentage is close to 50%. Likewise, another peculiarity of senior doctors for the increased risk of receiving complaints is the complexity of the cases they work with, as in some specialties there is a limitation in this regard depending on the degree and experience of the doctor.

At the opposite pole, the lower risk of receiving complaints among resident doctors may be related to the fact that they work under supervision, with this aspect sometimes being erroneously viewed as a lack of responsibility in the eyes of the population and sometimes in the eyes of resident doctors as well. Indeed, during their professional training, resident doctors can practice only under supervision, carrying out their activity in the field of the specialty within the limits of the competences corresponding to their training year, under the strict supervision and guidance of a specialist or senior doctor. However, this does not mean that resident doctors are immune and not responsible for the way they perform their duties, when they exceed their competencies, when they make decisions on their own or when they do not communicate properly with other members of the medical team [28].

Another relevant result of the present study was the link between the risk of complaints and the number of on-call shifts, doctors who perform more shifts being more likely to be complained about. This result can be interpreted in the context of overload and overnight work schedule. The Regulation on working time, organization and performance of on-call shifts in public medical institutions in Romania provides that “normal working time […] is 7 h on average per day, respectively 35 h on average per week”, being reduced by an hour for a series of specialties such as those in which the doctors perform postmortem examinations (pathology, forensic medicine) and those involving the risk of irradiation (e.g., radiology, radiotherapy, nuclear medicine) [29], but often this program is exceeded, doctors being forced to work over the schedule—either because of the shortage of doctors or because of the very large number of patients.

In order to ensure the permanence of medical assistance in hospitals, the doctors carry out an on-call shifts program, which in Romania starts after the usual working schedule and ends at the beginning of the working schedule of the next day, and on weekends and other official days off, the program for on-call shifts begins in the morning and ends the next day, after 24 h. Normally, after 24 h of work, doctors should rest, but often after on-call shifts doctors continue the regular schedule, so they may work up to 32 h without interruption during the week. There is no maximum number of on-call shifts allowed, the legislation in the field only specifying that it is forbidden to work two consecutive shifts [29]. Extrapolating, we can estimate that a doctor can perform a maximum of 15 on-call shifts in a month with 30 days. In the present survey, doctors reported 0 to 15 on-call shifts per month, with a mean of 2.26 (±2.78). Although the on-call schedule is not as demanding in all specialties, the shortage of doctors means that in many hospitals doctors have to perform a large number of on-call shifts, so they have little time to rest.

Similarly, a study conducted in Japan, where the normal work schedule is 40 h per week, showed that pediatricians often work over the working program, and 8% of them end up working more than 79 h a week, with a mean of 86.7 overworking hours each month and 32 consecutive hours of work when performing on-call shifts. Moreover, in Japan an overwork-related cause of death is recognized, which is known as karoshi [30].

Ensuring the continuity of health care services is an imperative of many health systems [31], even if the way it is done may differ from country to country: 24 h hospital shifts, home on-call shifts, shifts of 12 h, night shifts. However, regardless of how it is performed, the work schedule, night shift and extension of the regular work schedule can cause increased levels of stress and fatigue among physicians by interfering with their sleep schedule or sleep duration, can lead to decreased work performance, and may predispose them to chronic disease [31] or anxiety and depression [32]. Under these conditions, the risk of errors increases as well, endangering the proper care of the patient [31].

Luckhaupt et al. [33] reported that between 1985 and 1990 and later, between 2004 and 2007, there was an increase in the percentage of medical professionals who did not get enough sleep during the entire day (i.e., ≤6 h), from 28% to 32%.

The consequences of insufficient sleep and fatigue for medical practice can be important: the state of alertness decreases, periods of involuntary micro-sleep may occur (lasting several seconds, in which although the person has their eyes open, the brain is blocked and attention disappears), reaction time increases, the ability to concentrate, store and process new information decreases [31], and the skills are reduced [34]. Studies on resident doctors show that these negative effects of sleep deprivation can occur despite ambition, training and experience [31]. Additionally, the effects of a lack of proper rest can interfere with the ability to relate to and communicate with patients [31,35], with doctors becoming irritable and experiencing mood swings, as well as with the decision-making process, leading to a tendency to take too many risks in the activity [31]. A study on 301 anesthesiologists in New Zealand, which looked at the evaluation of fatigue errors caused by the work schedule, showed that 86% of participants confirmed the existence of errors in practice due to fatigue [36], and the study by Landrigan et al. [37] shows that during the extended work schedule of more than 24 h, the number of errors made by interns/trainees was 35.9% higher compared with the number of errors in the work schedule in shifts of maximum 16 h.

Moreover, doctors who have more shifts examine more patients, and there is again a higher risk of being exposed to complaints from patients [30].

The results of the current study show that out of the first six most risky specialties in relation to the number of doctors complained about in the total number of respondents in each specialty, five are major surgical specialties: plastic surgery, pediatric surgery, neurosurgery, general surgery and orthopedics & traumatology. In addition, when the categories of specialties (surgical, medical, pediatric, paraclinical-laboratory and family medicine) are compared, doctors working in the category of surgical specialties were the most complained about, and those working in family medicine received the fewest complaints. Malpractice complaints are incidents that surgeons frequently face in their careers [38], with numerous literature studies placing surgical specialties at the top of the list of specialties receiving the most complaints [1,13,20], along with obstetrics & gynecology [1,13]. In the study published by Jena et al. [38], in which the authors assessed the risk of complaints by specialty in the USA, the first three places are occupied by neurosurgery, cardiovascular surgery and general surgery. Taragin et al. [16] (New Jersey) identified neurosurgery as taking first place, along with orthopedics and obstetrics & gynecology, these specialists accumulating seven to nine times more complaints per year than psychiatry, the specialty least prone to complaints. Tibble et al. [20] ranks the first three places among the surgical specialties receiving complaints in Australia as neurosurgery, plastic surgery and orthopedic surgery, sustaining that they pose a higher risk compared with general surgery. In the study performed by Boyll et al. [27], of 129 plastic surgeons who were members of the American Society of Plastic and Aesthetic Surgery, nearly three-quarters stated that they had faced at least one complaint from patients. In Italy, between 1996 and 2000, the two specialties receiving the most complaints were orthopedics & traumatology and obstetrics & gynecology [2].

A possible explanation for the increased predisposition of surgeons to be complained about by patients is the intrinsic risk of the specialty [1,20], given the invasive nature of the treatment [39] and the generally high severity of the diseases they treat [20,40]. In addition, given that patients sometimes suffer from multiple comorbidities, and technological progress allows the use of increasingly advanced and complex procedures, the associated risks are directly proportional [41], but in turn, society is constantly changing and has increasing expectations, accepting these risks less and less [42]. The first place being occupied by neurosurgery in some studies can be explained by the small number of doctors working in this specialty [43]. In Romania, neurosurgeons are less than 1% of the total number of doctors (0.63%) [44], and in the group in the present study they represented 0.83%—of the 14 participating doctors, six had received complaints from patients. However, the higher risk for neurosurgeons also stems from special features, such as neurological complications resulting in functional disorders being difficult to accept by patients [42,45]. For plastic surgery, studies show that most complaints are related to patients’ dissatisfaction with the results of cosmetic interventions [27,46]. In this regard, some authors suggest that complaints arise due to poor and unclear information about the expectations that patients should have after cosmetic interventions [27,47]. In addition, studies that targeted only plastic surgeons showed a 2.5 times higher risk among doctors who focus on cosmetic interventions in their practice, compared with others [27].

To these explanations can be added the relatively poor relationship skills among surgeons, which predisposes them to poor communication with patients and consequently to complaints [20].

The present study indicates that family medicine is one of the specialties in which doctors receive the fewest complaints, which is partly consistent with the results obtained in a previous study [7]. Although there are studies in the literature that place a generally high risk for family medicine [48,49], the fact that this specialty received fewer complaints in the present and the previous studies is not a situation limited to Romania, as there are other authors who have obtained similar results [38]. What is, however, particular for Romania is the general context in which patients often avoid using the services of the family doctor, instead requesting in excess the emergency services—either by calling the unique emergency number 112, which involves driving an ambulance at the patient’s home, or by presenting directly to the emergency unit for conditions that could be resolved by the family doctor [50]. This preference of patients for the emergency services to the detriment of family doctors creates an imbalance in the health system, often resulting in doctors in the emergency services becoming overworked. As well as this, due to more urgent situations being prioritized, patients who abuse this service feel neglected, become irritated and thus the favorable context for a complaint is created [51].

Another characteristic of doctors prone to receiving complaints is their activity in regional or county hospitals. The explanations are multiple and related to those discussed above. Regional or county hospitals are large hospitals, which have superior competences, better equipment for diagnosis and treatment, thus having opportunities to care for patients with conditions of high complexity [52,53]. Thus, doctors are exposed to risk through the care provided to a large number of patients, who often have complex pathologies. In this way, the overloading of doctors reduces the time allotted to patients, with the subsequent dissatisfaction of the latter. Similar results were obtained by Hwang et al. [54], who found in their study that more than 70% of complaints concerned large medical centers and regional hospitals.

Fear of complaints as a factor that predisposes doctors to receiving complaints may be related to the fact that fear of complaints can cause the doctor to make decisions for their personal protection, which in turn can lead to complaints, for example by decreasing the patient’s adherence to treatment [55]. At the same time, other authors claim that requesting too many investigations could raise some questions about the competence of the doctor or the quality of care [21]. This could explain the result of the present study which shows that the greater fear of complaints—which induces changes in medical practice—predisposes doctors to complaints from patients. Nevertheless, there are studies that show that supplementation of investigations gives patients the impression that they are better cared for, without considering the fact that more care does not necessarily equate to better quality of care [56].

The results of the present study indicate there is a higher risk of receiving complaints for doctors whose life partner is a doctor with the same specialty. The relevance of this characteristic requires more in-depth analysis in future studies, as no other studies found in the literature focused specifically on this issue. Consequently, a comparison to corroborate our results was not possible in the present paper.

## 5. Reflections and Planning

Many of the characteristics found to compose the profile of the Romanian doctor prone to receiving complaints swing around the same core, i.e., doctor–patient communication. Therefore, specific activities aiming to improve this essential component of the medical practice may help to reduce the number of complaints.

Examples of such activities would be enhancement of awareness regarding this issue starting at the university level, with periodic updates throughout the doctor’s career through participation in training specifically dedicated to various issues connected to doctor–patient communication (e.g., manner in which to reveal the occurrence of a mistake or a complication, manner in which to hold discussions with different types of patients, how to address each patient when the waiting room is crowded, the limits of the new technology in medicine).

The results reflect the situation related to malpractice complaints in Romania. The fact that doctors at higher risk of receiving complaints are those in the category of surgical specialties (plastic surgery, pediatric surgery, neurosurgery, general surgery, orthopedics & traumatology) makes it necessary to study them further in depth, to identify specific risks and implicitly to implement targeted measures to prevent them.

In addition, we found that the specialty with the lowest risk is family medicine, a finding that could be explained by the under-use of the primary health care services to the detriment of emergency medical services, suggesting the need for measures aiming to improve the appropriate access of health services by patients.

The increased risk of being complained about among doctors who perform more on-call shifts raises an alarm about the risks of overload and suggests the need for collaboration between the relevant bodies to protect overworked doctors and implicitly patients requesting their services.

The results obtained are important for doctors, medical institutions and policy makers in order to implement new rules and practices to diminish the risk for malpractice complaints.

## 6. Strengths and Limitations of the Study

A significant strength of the present study is its pioneering character, being the first research in Romania that aims to analyze complaints regarding professional responsibility, being addressed directly to doctors, at the national level. Second, this study allowed the outlining of the profile of the Romanian doctor prone to receiving complaints from patients. In this way, the study highlighted common aspects shown by studies conducted in other countries, but also particular aspects explained by the characteristics of the medical practice in Romania and the organization of the medical system in this country.

A limitation of this study is the fact that the results concern doctors in Romania, so they cannot be generalized, or extrapolated to the situation of other countries. A second limitation could be related to the arbitrary choices of bins of the HL test.

## 7. Conclusions

The present study allowed us to outline the socio-demographic, professional and institutional characteristics of the Romanian doctor prone to being complained about by patients and, implicitly, highlighted aspects where interventions can be made at the national level to reduce the risk of malpractice complaints. The results outline the profile of the Romanian doctor prone to being complained about: male, senior doctor, from surgical specialties, who performs a larger number of on-call shifts, who works in regional or county hospitals, who has a higher level of fear of complaints and whose life partner is a doctor with the same specialty.

## Figures and Tables

**Table 1:** Socio-demographic characteristics and professional activity of the participants ^1^.

Characteristics	N (%)
**Gender**	
Male	488 (29.0)
Female	1196 (71.0)
**Marital status**	
Single	302 (17.9)
In a relationship	1382 (82.1)
**Life partner’s profession**	
Doctor, same specialty	96 (6.9)
Doctor, different specialty	352 (25.4)
Nurse	40 (2.8)
Other profession in the medical field	44 (3.1)
Other profession	850 (61.5)
**Children**	
No	543 (32.2)
Yes	1141 (67.8)
**Graduating institution**	
Private	50 (3.0)
Public	1634 (97.0)
**Medical specialty**	
Family medicine	321 (19.1)
Medical	692 (41.1)
Surgical	370 (22.0)
Pediatrics	123 (7.3)
Laboratory	178 (10.6)
**Area of activity**	
Urban	1522 (90.4)
Rural	88 (5.2)
Both	74 (4.4)
**Type of medical institution I**	
Public	749 (44.5)
Private	482 (28.6)
Both	453 (26.9)
**Type of medical institution II**	
Regional/county hospital	709 (42.1)
Municipality hospital	274 (16.2)
City hospital	101 (5.9)
Polyclinic/ambulatory	525 (31.1)
Private medical office	513 (30.4)
Private hospital/clinic	658 (39.0)
**Type of patients**	
Mostly women	260 (15.4)
Mostly men	62 (3.7)
Women and men equally	1362 (80.9)

^1^ Number of answers (N) and percentage (%).

**Table 2:** Results of the omnibus test for regression coefficients.

		Chi-Square	df	Sig.
Step 1	Step	261,043	22	0.000
	Block	261,043	22	0.000
	Model	261,043	22	0.000

**Table 3:** Hosmer–Lemeshow Test.

Step	Chi-Square	df	Sig.
1	7.606	8	0.473

**Table 4:** Variables in the Equation.

		B	S.E.	Wald	df	Sig.	Exp(B)	95% C.I. for EXP(B)	
								Lower	Upper
Step 1 ^a^	Gender (1)	−0.919	0.170	29.185	1	0.000	0.399	0.286	0.557
	Age	0.034	0.023	2.076	1	0.150	1.034	0.988	1.083
	Children (1)	−0.158	0.196	0.649	1	0.421	0.854	0.582	1.253
	Specialty category			20.477	4	0.000			
	Specialty category (1)	0.741	0.255	8.416	1	0.004	2.098	1.272	3.461
	Specialty category (2)	1.148	0.266	18.654	1	0,000	3.151	1.872	5.305
	Specialty category (3)	0.678	0.369	3.379	1	0.066	1.970	0.956	4.057
	Specialty category (4)	0.508	0.329	2.376	1	0.123	1.661	0.871	3.169
	Professional degree			26.542	2	0.000			
	Professional degree (1)	1.925	0.737	6.815	1	0.009	6.853	1.615	29.070
	Professional degree (2)	2.793	0.749	13.890	1	0.000	16.322	3.758	70.885
	Partner’s profession			8.988	5	0.110			
	Partner’s profession (1)	−0.810	0.300	7.273	1	0.007	0.445	0.247	0.802
	Partner’s profession (2)	−0.531	0.516	1.058	1	0.304	0.588	0.214	1.617
	Partner’s profession (3)	−0.345	0.324	1.136	1	0.286	0.708	0.375	1.336
	Partner’s profession (4)	−0.930	0.543	2.933	1	0.087	0.395	0.136	1.144
	Partner’s profession (5)	−0.593	0.282	4.413	1	0.036	0.553	0.318	0.961
	Fear of complaint			37.729	4	0.000			
	Fear of complaint (1)	−0.644	0.198	10.564	1	0.001	0.525	0.356	0.774
	Fear of complaint (2)	−0.880	0.264	11.108	1	0.001	0.415	0.247	0.696
	Fear of complaint (3)	−1.381	0.234	34.942	1	0.000	0.251	0.159	0.397
	Fear of complaint (4)	−1.306	0.415	9.899	1	0.002	0.271	0.120	0.611
	Seniority in medical system	0.000	0.022	0.000	1	0.995	1.000	0.958	1.043
	Number of on-call shifts monthly	0.062	0.027	5.220	1	0.022	1.064	1.009	1.122
	County/regional hospital (1)	0.338	0.166	4.145	1	0.042	1.403	1.013	1.943
	University teacher (1)	−0.045	0.201	0.049	1	0.825	0.956	0.645	1.419
	Constant	−4.635	1.039	19.882	1	0.000	0.010		

^a^ Variable(s) entered on step 1: gender, age, children, specialty category, professional degree, partner’s profession, fear of complaint, seniority in medical system, number of on-call shifts monthly, county/regional hospital, university teacher.

## Data Availability

Data are available, on request, from the corresponding author.
